# SAPO-1/Fas and sFas-L ratio, level of Bcl-2 and p53 as a predictors of multiple organ dysfunction syndrome in polytrauma

**DOI:** 10.1186/cc9837

**Published:** 2011-03-11

**Authors:** A Kiseleva, E Grigoryev, Y Churlyaev, A Radivilko

**Affiliations:** 1 Branch of the Institute of General Reanimatology, Novokuznetck, Russia; 2 Institute of Complex Problems of Cardiovascular Diseases, Kemerovo, Russia; 3 Medical Academy, Kemerovo, Russia

## Introduction

The objective was to determine the prognostic significance of serum markers of apoptosis in patients with polytrauma.

## Methods

The study included 34 male patients (38 ± 21 years old) with polytrauma. The severity of patients on admission according to the ISS scale was 35 ± 14, on a scale of APACHE II was 26 ± 6, and on the SOFA scale was 7 ± 4. We investigated the serum markers of apoptosis: sAPO-1/Fas (soluble Fas receptor, sFas), sFas-L (soluble Fas ligand), Bcl-2 and p53 (Bender MedSystems, Austria). Data are presented as mean ± standard deviation.

## Results

In patients with severe injury on the first day determined by the initial high level of sAPO-1/Fas (410.9 ± 89.7 pg/ml), which decreased on the second day, while remaining significantly above control values, the component for sAPO-1/Fas was 108 ± 12 pg/ml (*P *= 0.001). The level of sAPO-1/Fas increased, reaching a maximum on the fifth day (419.5 ± 94.5 pg/ml). The level of sFas-L was initially almost three times higher than the reference values at 48 ± 14 pg/ml, and on the third day rose in parallel to sAPO-1/Fas, reaching a maximum on the fifth day. In response to increased Fas-L, sFas is released. With increased expression of FasL and sFas lack of apoptosis leads to the development of multiple organ failure, and an excess of sFas massive death of lymphocytes may cause immunosuppression. The level of Bcl-2 in serum on the first day was significantly higher than in the control group (7.11 ± 5.55 ng/ml, *P *= 0.001) and amounted to 26.5 ± 6.3 ng/ml. On the fifth day there was a significant increase in the concentration of Bcl-2 to 39.8 ± 8.8 ng/ml, but by the seventh day the level of Bcl-2 decreased to 22.8 ± 4.3 ng/ml. Increased levels of p53 induced by hypoxia lead to increased concentrations of Bcl-2.

## Conclusions

The progressive development of multiple organ dys-function syndrome in polytrauma is associated with serum concentrations of sAPO-1/Fas and sFas-L ratio, Bcl-2 and p53.

